# Molecular Implications of Repeated Aggression: *Th*, *Dat1*, *Snca* and *Bdnf* Gene Expression in the VTA of Victorious Male Mice

**DOI:** 10.1371/journal.pone.0004190

**Published:** 2009-01-14

**Authors:** Natalia P. Bondar, Ul'yana A. Boyarskikh, Irina L. Kovalenko, Maxim L. Filipenko, Natalia N. Kudryavtseva

**Affiliations:** 1 Institute of Cytology and Genetics SD RAS, Novosibirsk, Russia; 2 Institute of Chemical Biology and Basic Medicine SD RAS, Novosibirsk, Russia; University of Parma, Italy

## Abstract

**Background:**

It is generally recognized that recurrent aggression can be the result of various psychiatric disorders. The aim of our study was to analyze the mRNA levels, in the ventral tegmental area (VTA) of the midbrain, of the genes that may possibly be associated with aggression consistently shown by male mice in special experimental settings.

**Methodology/Principal Findings:**

The genes were *Th*, *Dat1*, *Snca* and *Bdnf*; the male mice were a group of animals that had each won 20 daily encounters in succession and a group of animals that had the same winning track record followed by a no-fight period for 14 days. Increased *Th*, *Dat1* and *Snca* mRNA levels were in the fresh-from-the-fight group as compared to the controls. Increased *Th* and *Dat1* mRNA levels were in the no-fight winners as compared to the controls. Significant positive correlations were found between the level of aggression and *Th* and *Snca* mRNA levels.

**Conclusions:**

Repeated positive fighting experience enhances the expression of the *Th*, *Dat1* and *Snca* genes, which are associated with brain dopaminergic systems. The expression of the *Th* and *Dat1* genes stays enhanced for a long time.

## Introduction

It is generally recognized that recurrent aggression can be the result of various psychiatric disorders such as manic-depressive disorder, compulsive-obsessive disorder, epilepsy, posttraumatic stress, autism, Alzheimer's disease, attention deficit/hyperactivity disorder, mental retardation, schizophrenia, drug abuse etc [1]. According to many authors [2]–[6], aggression is rewarding for both laboratory rodents and humans and any positive reinforcement increases the propensity to behave aggressively. Rats and mice with the prior experience of social victories attack more frequently [6]–[11]. Mice with repeated positive fighting experience can develop violent behavior patterns [4], [12]. The same refers to humans: the individuals who have once displayed aggressive behavior tend to do so again [5].

It has been experimentally demonstrated that repeated aggression displayed by male mice leads the activation of brain dopaminergic systems. This activation was detected as elevated DOPAC (3,4-dihydroxyphenylacetic acid) levels or/and increased DOPAC/DA (dopamine) ratios in the olfactory bulbs, amygdala, hippocampus, nucleus accumbens, striatum and midbrain observed in the winners as compared to the controls [13], [14]. Reportedly, the dopaminergic systems can be activated in aggressive rats, as DA levels were elevated in the prefrontal cortex during and after fights [15]. A number of papers confirms the involvement of brain dopaminergic systems in the control of aggressive behavior [16].

The aim of our study was to analyze the mRNA levels of the *Th*, *Dat1*, *Snca* and *Bdnf* genes. These genes were chosen because of the role their products (proteins) have in the brain dopaminergic regulation: tyrosine hydroxylase (TH), which is the rate-limiting enzyme of DA synthesis; the dopamine transporter (DAT), which terminates the DA action on the postsynaptic membrane by rapidly removing it from the synaptic cleft via reuptake [17]–[19]; alpha-synuclein (SNCA), which plays a role in dopamine compartmentalization in the pre-synaptic terminals [20]–[21], vesicular dopamine storage, vesicular dopamine release into synapses, and dopamine re-uptake into the dopaminergic neurons [22]; the brain-derived neurotrophic factor (BDNF), which is associated with many diseases [23], [24]. We focused on the ventral tegmental area (VTA) of the midbrain containing the cell bodies of mesolimbic dopaminergic neurons, because mesolimbic dopaminergic projections from the VTA play an important role in the mediation of rewarding processes and are associated with many types of social behavior [25], [26].

The mRNA levels were analyzed in male mice that had a long positive fighting history (20 wins in daily agonistic interactions) and developed behavioral psychopathology, which included the demonstration of abnormal aggression, malignancy, strong hostility, pronounced anxiety, disturbances in social recognition, hyperactivity, stereotypic and hyperkinetic reactions etc [4]. The expression of these genes was also analyzed in a group of 20-time winners who afterwards had not been allowed to fight for 14 days referred to as “the period of aggression deprivation” or “the period of deprivation” throughout; such animals are special in that they are even more aggressive after than before this no-fight period [4]. The comparison of the levels of expression of these genes in the fight-deprived and fight-undeprived winners helps answer the question as to whether the levels of gene expression in the VTA of the “deprived” winners recovers to that in the controls.

## Materials and Methods

### Animals

Adult male mice of the C57BL/6J strain from a stock maintained in the Animal Facility of the Institute of Cytology and Genetics, SD RAS, (Novosibirsk, Russia) were used. The animals were housed under standard conditions (12∶12 h light/dark regime, switch-on at 8.00 a.m.; food (pellets) and water available *ad libitum*). Mice were weaned at one month of age and housed in groups of 8–10 in plastic cages (36×23×12 cm). Experiments were performed on mice 10–12 weeks of age. All procedures were in compliance with the European Communities Council Directive of November 24, 1986 (86/609/EEC).

### Winners

Aggressive behavior was induced using the sensory contact model [27]. Pairs of weight-matched animals were each placed in a steel cage (28×14×10 cm) bisected by a perforated transparent partition allowing the animals to see, hear and smell each other, but preventing physical contact. The animals were left undisturbed for two or three days to adapt to new housing conditions and sensory contact before they were exposed to encounters. In the second half of the light period, the lid was replaced by a transparent one and five minutes later the partition was removed for 10 minutes to encourage agonistic interactions. The superiority of one of the mice was firmly established within two or three encounters with the same opponent. The superior mouse would be attacking, biting and chasing another, who would be displaying only defensive behavior (sideways postures, upright postures, withdrawal, lying on the back or freezing). The duration of each fight was kept to three minutes, at which point the partition was pulled down. Each defeated mouse (loser) was exposed to the same winner for three days, while afterwards each loser was placed, once a day after the fight, in an unfamiliar cage with an unfamiliar winner behind the partition. Each victorious mouse (winner) remained in its original cage. This procedure was performed once a day for 20 days and yielded an equal number of winners and losers. The controls were animals that had been housed individually for five days. The rationale for this choice is that it gives the best trade-off between group housing and social isolation: five days is sufficient for group housing to no longer be a factor and insufficient for social isolation to become a factor [27].

**Figure 1:**
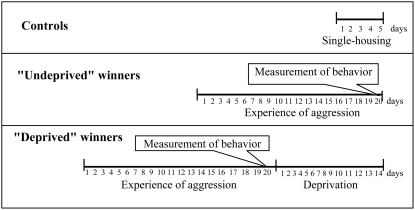
Protocol of the experiment. Detailed explanations are given in the text. Behavior in “undeprived” and “deprived” winners as recorded during their respective last encounter.

The design of the current experiment is presented in Figure 1. Three groups of animals were used. (1) Fight-undeprived winners: a group of mice that had each won 20 encounters in succession. (2) Fight-deprived winners: a group of 20-time winners who were allowed to live for 14 days after the last encounter. During this period, each of them shared a cage with a loser; the partition between their compartments being down at all times, to prevent encounters. (3). Controls: the mice that had been housed individually for five days before they were killed for scientific purposes. Each experimental group contained 7–11 animals.

### Behavioral study

Each winner was video recorded for 10 min during its last encounter (Figure 1) and the data were documented. Furthermore, we needed to know whether both groups of winners could be considered identical at the time each mouse won its last encounter. If they were, all the differences in gene expression between fight-deprived and fight-undeprived winners, or lack thereof, could solely be accounted for by deprivation. To find out, the groups were compared in terms of behavior.

The following were the behavioral domains analyzed. *1. Attacking*. Attacking, biting and chasing. *2. Aggressive grooming*. The winner mounts onto the loser's back, holds it down and spends much time licking and nibbling at the loser's scruff of the neck. The loser is wholly immobilized – or sometimes stretches out the neck and again freezes under the winner. 3. *Digging*. Digging up and scattering the sawdust on the loser's territory (kick-digs: pulling the sawdust forwards with the forepaws; push-digs: pushing the sawdust backwards with the hind paws). 4. *Self-grooming*. Body care activities (fur licking, head washing, nose washing).

The following were the behaviors measured. a. Latency to attacking; b. Total time spent on any of the four above listed activities; c. Number of events falling under any of the four above listed domains. If an animal did not attack or aggressively groom during the session, the latency to these events was assigned a duration of 600 s, which is how long the session lasted, and the other measures were assigned a value of zero. The total time spent attacking, aggressively grooming and digging was counted as a measure of *hostile behavior*.

All the mice were decapitated simultaneously (Figure 1). An important point is that the “undeprived” winners were decapitated 24 hours after the last encounter. Brains were removed and chilled rapidly on ice. The VTA was dissected according to the Mouse Brain Atlas [28] and sections were collected from 1.68 mm to −2.12 mm relative to bregma. Obtained tissue was rapidly frozen in liquid nitrogen and stored at −70°C until used.

### Total RNA extraction and reverse transcription

Total RNA was extracted from each individual brain tissue sample using the Chomczynski and Sacchi method [29] with modifications. Total RNA was quantified by measuring the absorbance at 260 nm. The integrity of total RNA was assessed by agarose gel electrophoresis. 1 µg of total RNA was used for cDNA synthesis by MoMLV reverse transcriptase (Biosan, Novosibirsk, Russia).

### Real-time quantitative PCR

Amplification was performed using an iQ5 iCycler (Bio-Rad, Hercules, CA, USA). *Th*, *Dat1*, *Bdnf*, β-actin (*Actb*), and cyclophilin (*Cphn*) mRNA levels were quantified by TaqMan real-time PCR. PCR was performed in a total volume of 25 µl containing an aliquot of the RT mixture, dNTPs, the appropriate concentrations of sense and anti-sense primers, a TaqMan probe, PCR buffer, and hot-start Taq DNA polymerase (Biosan, Novosibirsk, Russia). Amplification was run for 2 min at 96°C, followed by 37 cycles of 15 s at 96°C, 45 s at 61°C. Fluorescence was monitored for 10 s after the last cycle.

*Snca* mRNA levels were quantified by SybrGreenI real-time PCR in a total volume of 25 µl containing an aliquot of the RT mixture, dNTPs, the appropriate concentrations of the sense and anti-sense primers, Sybr Green I (Invitrogen), PCR buffer, and hot-start Taq DNA polymerase. Amplification was run for 3 min at 95°C, followed by 40 cycles of 10 s at 92°C, 6 s at 60°C, 6 s at 72°C and 10 s at 85°C. Fluorescence was monitored for 10 s after the last cycle. To check for the presence of non-specific PCR products or primer-dimers, a melting curve analysis was performed after the final PCR cycle.

Amplification efficiencies were calculated a relative standard curve derived from fourfold serial dilutions of pooled cDNA. In all cases, the amplification efficiency was higher than 85%. Each sample was PCR-amplified twice. RT-PCR results were quantified using the relative standard curve method. The level of expression of each gene was normalized to the mean level of expression of the *Actb* and *Cphn* genes.

The oligonucleotide primers and probes were designed using Beacon Designer 5.0 (PREMIER Biosoft International, USA). The PCR primer and probe sequences are shown in Table 1.

**Table 1:** Primer and probe sequences.

Genes	Primer and probe sequences	
***Bdnf***	sense	5′-ACTATGGTTATTTCATACTTCGGTT-3′
	anti-sense	5′-CCATTCACGCTCTCCAGA-3′
	probe	5′-FAM-CGTCCACGGACAAGGCAACTT-BHQ1-3′
***Dat1***	sense	5′- GTGTCCAGCAATTCAGTGAT-3′
	anti-sense	5′-TGACCACGACCACATACAGA-3′
	probe	5′- FAM-CCAGCATAGCCGCCAGTACAGG-BHQ1-3′
***Th***	sense	5′-TTGGATAAGTGTCACCACCTG-3′
	anti-sense	5′-TGGCTCACCCTGCTTGTA-3′
	probe	5′-R6G-TGACCCTGACCTGGACCTGGAC-BHQ1-3′
***Snca***	sense	5′-TGACAGCAGTCGCTCAGA-3′
	anti-sense	5′-CATGTCTTCCAGGATTCCTTC-3′
***Cphn***	sense	5′-GAGAACTTCATCCTAAAGCATACAG-3′
	anti-sense	5′-TCACCTTCCCAAAGACCA-3′
	probe	5′- TAMRA -CGTTGCCATCCAGCCATTCAG-BHQ2-3′
***Actb***	sense	5′- TCTTTGCAGCTCCTTCGTT -3′
	anti-sense	5′-CGATGGAGGGGAATACAG-3′
	probe	5′- ROX-CACACCCGCCACCAGTTCGC-BHQ2-3′

### Statistics

Statistical analysis was performed using the Kruskal-Wallis one-way analysis of variance (ANOVA) with factor groups. A post-hoc pair-wise comparison of the groups was made with the Mann-Whitney test (*U* test). Correlations were assessed using Spearman's rank correlation coefficient. We searched for correlations between the *Th*, *Dat1*, *Bdnf*, and *Snca* mRNA levels in each experimental group separately and in combination; each *Th*, *Dat1*, *Bdnf*, *Snca* mRNA level and each behavior (latency to first attack, the number of attacks, the total amount of time spent attacking) in the “undeprived” winners; post-deprivation *Th*, *Dat1*, *Bdnf*, and *Snca* mRNA levels and pre-deprivation behavioral parameters in the “deprived” winners. The statistical significance was set at P≤0.05; the tendency level was set at 0.05<P<0.1.

## Results

No differences were found between the “undeprived” and the “deprived” group in any of the individual or social behaviors measured after the respective 20-day periods of agonistic interactions (P>0.05, Table 2). Therefore, behaviorally, both groups could be considered identical.

**Table 2:** Behavioral data from winners in the “undeprived” and the “deprived” group during their respective last encounter.

Behavioral parameters		“Undeprived” winners	“Deprived” winners	Mann-Whitney test
**Attacks**	Latency, s	41.7±15.0	68.9±39.5	U = 27.0; NS
	Number	15.3±3.1	12.0±2.8	U = 34.5; NS
	Total time, s	81.4±20.8	53.4±12.2	U = 29.0; NS
**Aggressive grooming**	Number	0.2±0.2	0.9±0.9	U = 36.0; NS
	Total time, s	3.2±3.2	9.4±9.4	U = 36.0; NS
**Diggings**	Number	10.2±1.7	9.9±1.2	U = 38.0; NS
	Total time, s	37.9±7.9	43.6±3.0	U = 28.0; NS
**Total time of hostile behavior**		122.5±18.2	106.4±10.9	U = 33.0; NS
**Self-grooming**	Number	4.5±1.1	7.1±1.8	U = 26.5; NS
	Total time, s	10.2±2.1	13.3±3.5	U = 33.0; NS
**Number of animals**		11	7	

Kruskal-Wallis analysis revealed a significant influence of the factor groups on the mRNA level of *Th* [H(2,24) = 7.11, P<0.029] and *Dat1* [H(2,25) = 6.45, P<0.040]. The influence of the factor groups on the mRNA level of the *Snca* gene was not definitely significant, but strongly suggestive [H(2,26) = 5.80, P<0.055]. There was no significant influence of the factor groups on the expression of the *Bdnf* gene [H(2,24) = 0.16, NS].

**Figure 2:**
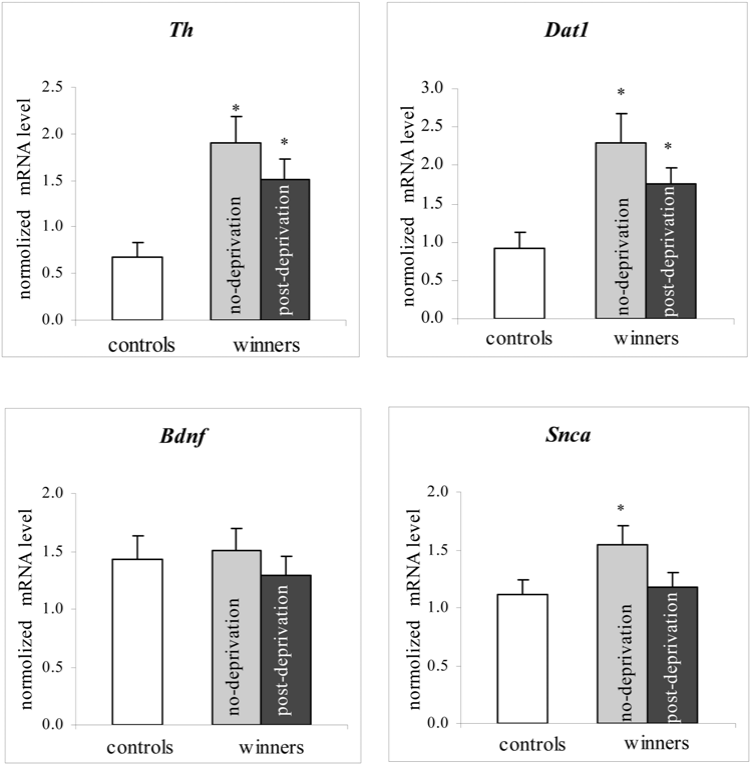
The normalized *Th*, *Dat1*, *Snca* and *Bdnf* mRNA levels in the VTA of the controls, “undeprived” and “deprived” winners. * - P<0.05 vs the controls (Mann-Whitney test).

Based on the Mann-Whitney test (Figure 2), the “undeprived” winners had increased mRNA levels of *Th* (U = 10; P<0.021), *Dat1* (U = 13; P<0.031) and *Snca* (U = 16; P<0.028) as compared to the respective levels in the controls; the “deprived” winners had increased mRNA levels of *Th* (U = 5; P<0.022) and *Dat1* (U = 5; P<0.022) as compared to the respective levels in the controls; there was no difference between the “undeprived” and the “deprived” group in the mRNA level of *Th* (U = 29; NS) or *Dat1* (U = 32; NS).

**Figure 3:**
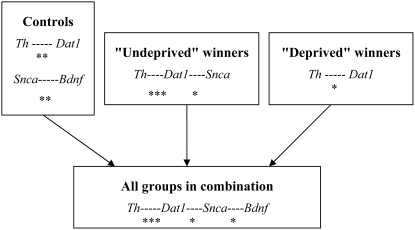
Significant correlations between the mRNA levels of the *Th*, *Dat1*, *Snca* and *Bdnf* genes in the VTA of the control, the “undeprived” and the “deprived” winners and all groups in combinations. Positive correlations: * - P<0.05; ** - P<0.01; *** - P<0.001; Spearman's rank correlation coefficient.

Based on Spearman's rank correlation coefficient, there were significant positive correlations between the mRNA levels of the following genes: *Th* and *Dat1* (R = 0.943, P<0.005), *Bdnf* and *Snca* (R = 0.893, P<0.007) in the controls; *Th* and *Dat1* (R = 0.891, P<0.001), *Dat1* and *Snca* (R = 0.636, P<0.026) in the “undeprived” winners; *Th* and *Dat1* (R = 0.857, P<0.014) in the “deprived” winners; *Th* and *Dat1* (R = 0.940, P<0.001), *Dat1* and *Snca* (R = 0.456, P<0.022), *Snca* and *Bdnf* (R = 0.479, P<0.018) using pooled data from all the groups (Figure 3).

**Figure 4:**
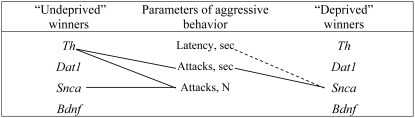
Significant correlations between the mRNA levels of the *Th*, *Dat1*, and *Snca* genes in the VTA of the “undeprived” and the “deprived” winners and the parameters of aggressive behavior during the 20^st^ confrontation. Solid lines – positive correlation; dotted line – negative correlations, P<0.05, Spearman's rank correlation coefficient.

Significant positive correlations were found between the *Th* mRNA level and the number of attacks (R = 0.607, P<0.047), the *Th* mRNA level and the total time spent attacking (R = 0.655, P<0.029) and the *Snca* mRNA level and the number of attacks (R = 0.699, P<0.017) in the “undeprived” winners; the *Snca* mRNA level and the total time spent attacking (R = 0.821, P<0.023), a negative correlation was found between the *Snca* mRNA level and the latency to first attack (R = −0.964, P<0.001) in the “deprived” winners (Figure 4). Other correlations failed to reach significance.

## Discussion

This experiment demonstrated an increase of the *Th* and *Dat1* mRNA levels in the VTA of C57BL/6J mice, each of whom won 20 encounters in succession (similar results had previously been obtained from CBA/Lac mice, each of whom won 10 encounters in succession [30]). Thus, a chronic manifestation of aggression, which is accompanied by the activation of the brain dopaminergic systems [13], [14], enhances the expression of the *Th* and *Dat1* genes, whose products are responsible for the synthesis and inactivation of DA, respectively. The increase of *Snca* expression, even though suggestive, may represent a feedback mechanism of DA re-uptake inhibition, which provides increased DA levels in the synaptic cleft under the influence of repeated aggression. No change in *Bdnf* expression was revealed in the winners. However, the expression of some genes may increase rapidly and decrease abruptly, while that of other genes changes more gradually [31]. As Miczek and the co-workers report [32], continuous subordination stress leads to significantly decreased levels of BDNF protein in the VTA compared to control levels, whereas intermittent social defeat stress episodes result in increased BDNF protein levels. Thus, the lack of changes in *Bdnf* mRNA levels in the winners could be explained by transient (dynamic) changes of gene expression shown, for example, for the genes of kappa-opioid receptors [33], [34], mu-opioid receptors [35], [36], and proenkephalin [37] in some brain areas in response to exposure to the experimental settings. If this explanation is correct, we cannot completely exclude the involvement of *Bdnf* in the mechanisms underlying repeated aggression. This expectation is supported by the presence of a positive functional correlation between the *Bdnf* and *Snca* mRNA levels.

In the “deprived” winners, *Th* and *Dat1* expression was still enhanced: the respective mRNA levels differed significantly from those in the control mice and did not from those in the “undeprived” winners. On the one hand, it is possible that living close to a male behind the perforated transparent partition alerts the winner and makes it more aggressive even in the no-fighting settings. Another interpretation could be that once the level of expression of these genes was enhanced due to repeated aggression, there might be molecular mechanisms in place to keep these levels enhanced, no matter which settings. The fact that the “deprived” winners are more aggressive than the “undeprived” winners [4] is, if nothing else, amusing. It is possible that the reason for enhanced aggression is the accumulation of DA due to an enhanced level of expression of the *Th* gene, which codes for TH, the key enzyme in DA synthesis. The *Snca* mRNA level in the “deprived” winners did not differ from that in the controls.

Significant positive correlations were found between *Th* and *Dat1* mRNA levels in the VTA within each of the experimental groups (the controls, the “undeprived” winners and the “deprived” winners), which suggests a close relationship between dopamine synthesis and inactivation, possibly as a result of overlapping of *Th* and *Dat1* mRNA-positive dopaminergic neurons [17]. The fact itself that there are positive correlations between *Th* and *Dat1* mRNA levels in the VTA is not surprising, because it is obvious that the products of these genes (TH and DAT proteins) are involved in dopaminergic mediation in brain. It is well known that the neurochemical regulation of neurotransmitters metabolism includes feedback mechanisms. Our data provide evidence that the *Th* and *Dat1* are part of these mechanisms. The reason for this correlative relationship might be the common molecular mechanisms of transcriptional regulation of these genes. For example, Nurr1 increases the transcriptional activity of both *Th* and *Dat1* promoters [38], [39]. A significant positive correlation between mRNA levels of the *Snca* and *Bdnf* genes was found in the control animals. It is possible that, in intact animals, the transcription factors that regulate the *Th* and *Dat1* genes are other than those that regulate the *Snca* and *Bdnf* genes. A positive correlation between mRNA levels of the *Dat1* and *Snca* genes was found in the “undeprived”, but not in the “deprived” winners. The *Snca* mRNA level in the “deprived” winners showed a tendency to recover to the control level.

Pooled data from all the experimental groups (the controls, the “undeprived” winners, the “deprived” winners) revealed an association of mRNA levels in the following succession: *Th----Dat1---Snca----Bdnf*. However, the intrinsic molecular mechanisms responsible for the functional association that exists between the experience of behaving aggressively, *Th*, *Dat1*, *Snca* and *Bdnf* expression and the implications of neurochemical events unfolding in the winners' brains have yet to be revealed.

Thus, a chronic manifestation of aggression, which leads to the activation of the brain dopaminergic systems, enhances the expression of the *Th* and *Dat1* genes, whose proteins are responsible for the synthesis and inactivation of DA, respectively. Mesolimbic dopaminergic projections from the VTA play an important role in the mediation of rewarding processes [reviews 25], [26], [40]. It is therefore possible that the observed changes of gene expression in the winners' VTA result from experiencing positive emotions over social victories [4]. Because consistent defeat leads to the activation of the serotonergic system and negative emotions [41], the lack of significant changes in *Th* and *Dat1* expression in the losers' VTA demonstrated previously [30] lends support to this possibility.

Interestingly, the winners' level of aggression measured as the latency to first attack, the number of attacks and the total time spent attacking is correlated with the *Th* and *Snca* mRNA levels in the VTA. It is therefore possible that the higher the level of aggression they display during encounters, the higher the level of *Th* and *Snca* gene expression in their brain.

The data obtained so far strongly support the statement that the *Th*, *Dat1* and *Snca* genes in the VTA are involved in the mechanisms of repeated aggression. It is most likely that these genes have a role in rewarding processes, which can directly underlie the motivation to behave aggressively again. However, these data cannot help answer the question as to whether an increase in the expression of these genes in the VTA is specific for aggressive behavior pathology developed due to repeated aggression and demonstrated in our behavioral experiments. Other neurotransmitter systems, too, may be factors; for example, the opioidergic or serotonergic systems, which were found altered in 20-time winners [4].

There is ample experimental evidence supporting the hypothesis that there are many genes that can change their functional state due to agonistic interactions [23], [33], [42]–[52]. Repeated exposure to social confrontations and social stress has been shown to be able to develop pathological states (depression, anxiety, abnormal aggression) in animals [4], [23], [41]. It is gradually becoming clear that the development of psychoemotional disorders leads to changes in the transcriptional state of a set of genes, which makes it possible to track changes in gene functioning and to look for possibilities of their pharmacological correction. If this is as it seems to be, we should think of a new-generation therapy that should prevent gene expression from being affected by psychopathogenic factors.
